# Plasmablast Storms: Microbial Drivers of Acute and Chronic Autoimmune Flares

**DOI:** 10.3390/microorganisms14010152

**Published:** 2026-01-09

**Authors:** Muhammad Soyfoo, Julie Sarrand

**Affiliations:** Department of Rheumatology, Hôpital Erasme, Université Libre de Bruxelles, 1070 Brussels, Belgium; julie.sarrand@ulb.be

**Keywords:** plasmablasts, autoimmunity, Epstein–Barr virus, cytomegalovirus, SARS-CoV-2, type I interferon, Toll-like receptors, dysbiosis, microbiome, extrafollicular response, epitope spreading, autoantibodies

## Abstract

Autoimmune flares are often accompanied by abrupt surges of circulating plasmablasts—short-lived, high-output antibody-secreting cells generated through extrafollicular B-cell activation in response to microbial cues. Three categories of microbial input appear to repeatedly trigger these “plasmablast storms”: latent herpesvirus reactivations (Epstein–Barr virus, cytomegalovirus, human herpesvirus-6, varicella–zoster virus), acute respiratory or gastrointestinal infections including SARS-CoV-2, and chronic oral or gut dysbiosis. Although biologically distinct, these stimuli converge on innate sensing pathways driven by pathogen-associated molecular patterns such as unmethylated CpG DNA, single-stranded RNA, lipopolysaccharide, and bacterial lipoglycans. Through Toll-like receptors and type I interferon signalling, microbial signatures accelerate class switching, amplify inflammatory cytokine milieus, and lower B-cell activation thresholds, enabling rapid plasmablast mobilisation. Dysbiosis further maintains B cells in a hyper-responsive state by disrupting mucosal homeostasis and altering microbial metabolite profiles, thereby reducing the stimulus required to trigger plasmablast bursts. Once generated, these waves of oligoclonal plasmablasts home to inflamed tissues, where chemokine and adhesion landscapes shape their retention during flares. Emerging evidence suggests that such episodic plasmablast expansions promote autoantibody diversification, somatic hypermutation, and epitope spreading, progressively eroding tolerance. This review synthesizes these insights into a unified model in which infections and dysbiosis promote microbe-licensed plasmablast storms that influence the tempo and severity of autoimmune disease.

## 1. Introduction

The clinical course of systemic autoimmune diseases is characterised by periods of relative quiescence punctuated by acute flares, episodes of intensified inflammation that drive cumulative organ damage and shape long-term prognosis [1,2]. Although the triggers for these flares remain incompletely understood, converging evidence implicates microbial exposures as key precipitants. Infections, viral reactivations, and perturbations in commensal microbiota have long been associated with disease onset and exacerbation in conditions ranging from systemic lupus erythematosus (SLE) to rheumatoid arthritis (RA), Sjogren’s syndrome, and inflammatory myopathies [3,4,5,6].

Three distinct categories of microbial input contribute to autoimmune flares, each operating through overlapping but mechanistically distinguishable pathways. First, latent herpesvirus reactivations, particularly Epstein–Barr virus (EBV), cytomegalovirus (CMV), human herpesvirus 6 (HHV-6), and varicella-zoster virus (VZV), periodically emerge from dormancy to engage innate sensors and create inflammatory milieus permissive for autoreactive B-cell expansion [7,8]. Second, acute respiratory and gastrointestinal infections, including influenza and SARS-CoV-2, trigger important plasmablast responses that can include autoreactive clones [9,10]. Third, chronic microbiome dysbiosis at oral and gut barrier sites generates inflammatory signals that keep B cells poised near activation thresholds, lowering the stimulus required for full-blown plasmablast storms [11,12]. These three inputs, overt infection, subclinical reactivation, and dysbiosis, represent mechanistically distinct but convergent drivers of the plasmablast storm cycle.

It is important to acknowledge that plasmablast storms represent one of several interconnected mechanisms driving autoimmune flares, rather than the unique pathway. Neutrophil activation with release of neutrophil extracellular traps (NETosis), complement cascade amplification, and stromal cell inflammatory loops all contribute to flare pathophysiology and may operate independently of/or in concert with B-cell responses [13,14]. Nevertheless, the plasmablast storm model provides a particularly tractable framework for understanding how discrete microbial events translate into humoral autoimmune exacerbations.

**Figure 1:**
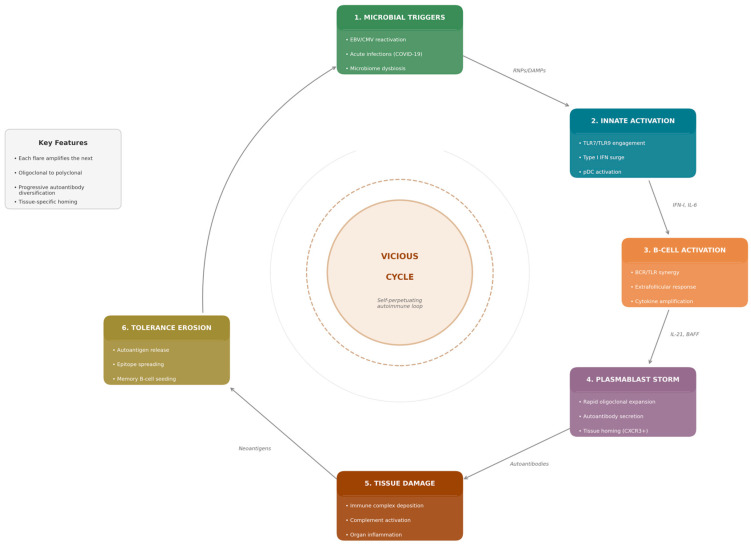
The plasmablast storm cycle in autoimmune disease. Three categories of microbial input, latent herpesvirus reactivation (EBV, CMV), acute infection (SARS-CoV-2, influenza), and chronic microbiome dysbiosis, activate innate sensing pathways through distinct PAMPs (CpG DNA, ssRNA, LPS, lipoglycans). TLR engagement and type I IFN production create inflammatory contexts that license extrafollicular B-cell responses. Rapid plasmablast expansion produces autoantibodies that drive tissue damage through immune complex deposition and complement activation. Tissue damage releases neoantigens, promoting epitope spreading and memory B-cell seeding. This tolerance erosion creates a self-perpetuating vicious cycle where each flare amplifies subsequent responses. Chronic dysbiosis maintains B cells near activation thresholds, lowering the stimulus required for discrete triggers to drive full plasmablast storms.

A unifying theme is the central role of plasmablasts—rapidly proliferating, short-lived antibody-secreting cells—in bridging these diverse microbial encounters with autoimmune pathology [15,16]. Unlike long-lived plasma cells that maintain stable antibody titres over years, plasmablasts represent an acute-phase response: they expand explosively within days of antigenic challenge and can infiltrate inflamed tissues [17,18]. This review synthesises current understanding of how microbial signals orchestrate plasmablast responses that fuel autoimmune flares (Figure 1).

## 2. Plasmablast Biology and Extrafollicular Responses

### 2.1. Defining Plasmablasts: Phenotype and Function

Plasmablasts occupy a transitional position in B-cell differentiation. Phenotypically, human plasmablasts are typically defined as CD19^low/+^CD20^−^CD27^high^CD38^high^ cells that retain proliferative capacity while actively secreting immunoglobulin [19,20]. The transient nature of plasmablasts, with circulating half-lives measured in days to weeks, belies their outsized impact on acute immune responses [21].

Recent single-cell analyses have revealed substantial heterogeneity within plasmablast populations. Transcriptomic profiling identifies subsets with distinct functional properties, including cells with enhanced inflammatory cytokine production, differential chemokine receptor expression, and varying degrees of commitment to the plasma cell fate [22,23].

Emerging evidence suggests that different microbial triggers may preferentially induce distinct plasmablast subgroups. Viral triggers, particularly EBV and SARS-CoV-2, appear to preferentially expand ISG^high^ plasmablasts characterised by strong interferon-signature gene expression and CXCR3^+^ tissue-homing phenotypes [24]. In contrast, bacterial stimuli engaging TLR2/TLR4 pathways may favour IL-6-driven, ISG^low^ plasmablasts with distinct metabolic profiles. Chronic dysbiosis has been associated with ‘exhausted’ or ‘atypical’ plasmablast phenotypes showing altered survival signals and reduced antibody secretion capacity [25]. Whether these microbe-specific signatures can be exploited diagnostically or therapeutically remains an active area of investigation requiring systematic single-cell comparisons across trigger types.

### 2.2. Extrafollicular Versus Germinal Centre Pathways

B-cell responses can proceed through two distinct pathways. The germinal centre (GC) reaction represents the classical route to high-affinity, class-switched, long-lived humoral immunity, unfolding over weeks [26]. In contrast, extrafollicular (EF) responses generate plasmablasts rapidly, within 3–5 days of antigen encounter [27,28]. The EF pathway can operate with reduced T-cell help, relying instead on innate signals including TLR ligands and type I interferons [29]. Critically, EF responses are subject to less stringent tolerance checkpoints, rendering them susceptible to autoreactive output. In SLE and other systemic autoimmune diseases, EF responses are pathologically expanded at the expense of GC reactions [30,31]. The dominance of EF responses in active autoimmune disease likely reflects the inflammatory cytokine milieu, which favours rapid plasmablast generation over the more deliberate GC pathway.

### 2.3. Why Plasmablasts, Not Germinal Centre B Cells, React to Microbial Triggers

The striking sensitivity of extrafollicular plasmablast responses to microbial triggers reflects evolutionary pressures that shaped humoral immunity. EF responses evolved as a rapid-reaction force against acute infections, providing antibody within days when waiting weeks for GC-derived high-affinity responses would prove fatal [32]. This evolutionary imperative for speed necessitated relaxed tolerance checkpoints: EF B cells can differentiate into plasmablasts with minimal T-cell help, relying instead on innate signals—particularly TLR7 and TLR9 engagement by microbial nucleic acids—to license differentiation [33].

Viruses uniquely favour EF over GC responses because the inflammatory milieu they induce—dominated by type I interferons and IL-6—actively promotes plasmablast differentiation while suppressing GC formation [34]. IFN-I inhibits Tfh cell development and GC B-cell survival while simultaneously driving IRF4 and BLIMP-1 expression that commits B cells to the plasmablast fate. In autoimmune disease, viral reactivations create inflammatory contexts that repeatedly channel B-cell responses through tolerance-relaxed EF pathways.

### 2.4. Transcriptional Control of Plasmablast Differentiation

The differentiation of activated B cells into plasmablasts requires coordinate changes in transcription factor expression. Central to this process is BLIMP-1 (encoded by PRDM1), a transcriptional repressor that silences genes required for B-cell identity. IRF4 cooperates with BLIMP-1 to promote plasma cell differentiation [35,36]. The cytokine environment profoundly influences the balance between these transcription factors. Type I interferons, IL-6, and IL-21 all promote IRF4 and BLIMP-1 expression through STAT-dependent mechanisms [37,38]. Microbial triggers amplify these transcriptional programmes by generating the precise cytokine signatures that tilt this balance toward plasmablast commitment. Infection-associated cytokine signatures thus create transcriptional contexts that favour rapid plasmablast differentiation over alternative B-cell fates.

### 2.5. Operational Definition of Plasmablast Storms

To enhance the translational value of the plasmablast storm concept, we propose provisional operational criteria modelled on established definitions for cytokine storm syndromes [39]. These criteria require prospective validation but provide a framework for clinical and research applications:

Quantitative threshold: Circulating plasmablasts exceeding 3–5% of total B cells (defined as CD19^low^CD27^high^CD38^high^ by flow cytometry), based on studies correlating this threshold with active SLE nephritis and disease flares [40].

Temporal dynamics: Rapid expansion (greater than 2-fold increase within 7–14 days) from patient baseline, distinguishing acute storms from chronically elevated plasmablast frequencies.

Clonality assessment: Oligoclonal dominance, operationally defined as the top 3 expanded clones representing greater than 30% of the plasmablast repertoire by BCR sequencing, indicating antigen-driven rather than polyclonal expansion.

Functional correlate: Concurrent rise in disease-relevant autoantibodies (anti-dsDNA, ACPA, anti-Ro/La) or validated clinical flare markers (e.g., SLEDAI increase ≥4 points).

We acknowledge that these thresholds derive primarily from SLE studies and may require disease-specific calibration. Standardisation of flow cytometry panels and BCR sequencing protocols across centres represents a prerequisite for multi-site validation. Nevertheless, establishing quantitative criteria transforms ‘plasmablast storms’ from a descriptive metaphor into a measurable entity amenable to biomarker development and therapeutic targeting.

## 3. Microbial Triggers of Plasmablast Storms

### 3.1. Epstein–Barr Virus: The Prototypical Trigger

EBV stands as the most extensively studied viral trigger for systemic autoimmunity [41,42]. Prospective studies have shown that EBV infection precedes and predicts the development of multiple sclerosis, with a 32-fold increased risk following infectious mononucleosis [43].

EBV occupies a unique position among plasmablast storm triggers because it is both a direct B-cell tropic virus, establishing latency specifically within memory B cells, and a potent amplifier of bystander innate sensing through release of immunostimulatory nucleic acids during reactivation [44]. This dual capacity—to directly manipulate infected B cells while simultaneously creating systemic inflammatory contexts—explains why EBV is so central to plasmablast biology in autoimmune disease.

During periodic viral reactivations, EBV-infected B cells undergo lytic replication and release viral particles containing unmethylated CpG DNA and small RNAs that engage TLR9 and TLR7 in plasmacytoid dendritic cells and B cells [45]. Studies in lupus patients demonstrate that EBV viral loads correlate with disease activity and that reactivation episodes precede clinical flares [46,47].

### 3.2. Cytomegalovirus and Other Herpesviruses

CMV contributes to autoimmune pathology through mechanisms distinct from EBV. Whereas EBV directly infects B cells, CMV’s primary latent reservoir is myeloid lineage cells. A distinctive feature of CMV infection is its profound impact on T-cell homeostasis, driving expansion of CD4^+^CD28^null^ T cells with features of immunosenescence [48].

CMV reactivation during immunosuppression or physiological stress releases viral particles containing immunostimulatory DNA that engages TLR9, potentially licensing bystander B-cell activation [8]. Molecular mimicry between CMV antigens and self-proteins, including phospholipids, has been proposed as a mechanism linking CMV infection to antiphospholipid antibody production [49]. Unlike EBV’s direct B-cell effects, CMV’s contribution to plasmablast storms operates primarily through T-cell-mediated pathways and innate immune activation.

#### 3.2.1. Human Herpesvirus 6

HHV-6 (comprising HHV-6A and HHV-6B species) infects over 90% of humans by age two and establishes latency in multiple cell types, including T cells, monocytes, and neural cells [50]. HHV-6 has been implicated in several autoimmune disorders, most notably multiple sclerosis, but also autoimmune connective tissue diseases and Hashimoto’s thyroiditis [51].

HHV-6A demonstrates particular neurotropism and has been detected in active MS plaques, with elevated anti-HHV-6 antibody titres in MS patients compared to controls [52]. The virus can trigger autoimmunity through several mechanisms: molecular mimicry between viral and myelin basic protein epitopes, bystander activation through lytic infection releasing sequestered autoantigens, and aberrant CD46 signalling that promotes IL-17-driven inflammation [53]. HHV-6 reactivation has been associated with flares of autoimmune connective tissue diseases, although direct causality remains to be established [54].

From a plasmablast storm perspective, HHV-6 reactivation provides type I interferon-inducing stimuli through TLR9 engagement by viral DNA, potentially lowering B-cell activation thresholds in genetically susceptible individuals.

#### 3.2.2. Varicella-Zoster Virus

VZV occupies a unique position among herpesviruses as the only human virus definitively shown to productively infect arterial walls and cause vasculopathy [55]. After primary varicella infection, VZV establishes latency in sensory ganglia along the entire neuraxis. Reactivation as herpes zoster can be followed by VZV vasculopathy, initially recognised in cerebral arteries but now known to affect extracranial vessels including temporal arteries.

The discovery of VZV antigen in temporal arteries of patients with giant cell arteritis (GCA) has generated intense interest in viral triggers for large-vessel vasculitis [56]. VZV has been detected in 73% of GCA-positive temporal artery biopsies versus 22% of pathologically normal biopsies in some studies [57]. The proposed mechanism involves transaxonal spread of reactivated virus from ganglionic neurons to arterial adventitia, followed by transmural infection that triggers granulomatous inflammation. However, inconsistent findings across studies and failure to reproduce positive results in some cohorts indicate that VZV is unlikely to explain all GCA cases [56].

For plasmablast biology, VZV reactivation provides a model of how periodic herpesvirus emergence from latency can trigger localised inflammatory responses with potential for bystander autoimmune activation.

#### 3.2.3. Herpesvirus Synergy and Co-Reactivation

Clinical and experimental evidence suggests that multiple herpesviruses may reactivate simultaneously during periods of immune stress, potentially amplifying inflammatory signals [58]. EBV-CMV co-infection has been associated with more severe autoimmune manifestations than either virus alone. The concept of a ‘herpesvirus burden’—cumulative exposure and reactivation frequency across the herpesvirus family—may help explain individual variation in autoimmune susceptibility.

From the plasmablast storm perspective, herpesvirus co-reactivation provides multiple simultaneous innate sensing inputs through TLR7 (viral RNA) and TLR9 (viral DNA), creating particularly robust type I interferon responses that drive extrafollicular B-cell differentiation.

### 3.3. SARS-CoV-2 and Acute Respiratory Infections

SARS-CoV-2 infection induces profound plasmablast expansion, sometimes exceeding 30% of circulating B cells in severe cases, with evidence of extrafollicular pathway dominance [59]. Single-cell analyses reveal oligoclonal plasmablast expansions with autoreactive specificities, including anti-interferon, anti-phospholipid, and anti-nuclear antibodies [60].

The COVID-19 pandemic served as a large-scale natural experiment validating the plasmablast storm model beyond classical rheumatic disease. The observations that severe infection drives extrafollicular B-cell responses with autoreactive output parallel findings in SLE [61]. Recent studies of long COVID have demonstrated persistence of autoreactive plasmablast clones and autoantibodies months after acute infection, suggesting that intense innate activation can establish durable autoimmune memory in susceptible individuals [62,63].

### 3.4. Mucosal Infections and the Mouth-to-Flare Connection

Periodontal pathogens, notably Porphyromonas gingivalis, have been mechanistically linked to RA through their capacity to citrullinate host proteins via PAD enzymes, generating neoepitopes recognised by ACPA [64,65]. The ‘mouth-to-flare’ model positions periodontal infection as an active, ongoing source of antigenic stimulation that can repeatedly trigger systemic plasmablast responses [66].

## 4. Innate Sensing Pathways Licensing Plasmablast Responses

**Figure 2:**
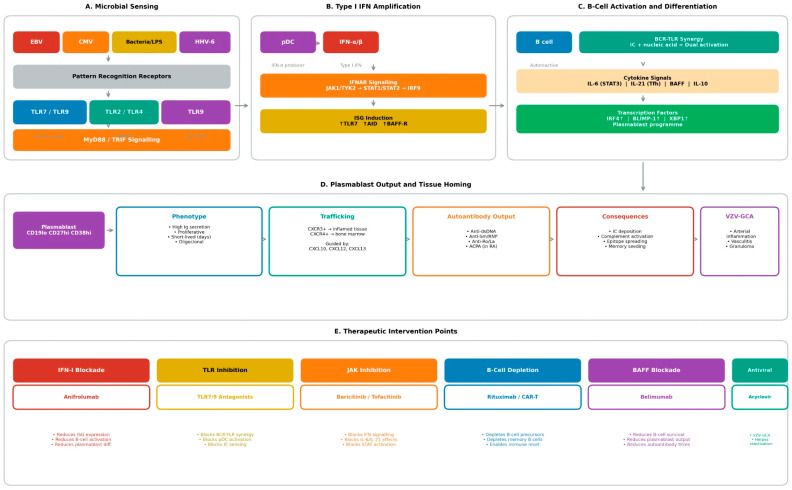
Molecular pathways licensing plasmablast differentiation. (**A**) Microbial sensing: Viral (EBV, CMV) and bacterial products engage endosomal TLR7/9 (detecting ssRNA and CpG DNA) and surface TLR2/4 (detecting LPS, lipoglycans, lipopeptides), initiating MyD88/TRIF signalling. (**B**) Type I IFN amplification: pDCs produce IFN-alpha/beta upon TLR engagement; IFNAR signalling through JAK-STAT induces ISG expression including TLR7 upregulation, creating positive feedback. (**C**) B-cell activation: BCR-TLR synergy from internalised immune complexes or direct microbial products, combined with cytokine signals (IL-6, IL-21, BAFF), drives transcription factor programmes (IRF4, BLIMP-1, XBP1). Metabolite depletion (reduced SCFAs) removes epigenetic restraints. (**D**) Plasmablast output: Short-lived, proliferative cells with oligoclonal expansion; tissue homing via CXCR3 (to inflamed tissue) or CXCR4 (to bone marrow); autoantibody production drives pathology. (**E**) Therapeutic targets at each node of the pathway.

The molecular mechanisms through which microbial signals license plasmablast differentiation centre on innate sensing pathways, particularly Toll-like receptor signalling and type I interferon production (Figure 2). These pathways integrate detection of specific pathogen-associated molecular patterns (PAMPs) with B-cell activation, creating permissive contexts for extrafollicular responses that bypass normal tolerance checkpoints.

### 4.1. Toll-like Receptor Signalling and Pathogen-Associated Molecular Patterns

TLRs represent the frontline sensors through which B cells detect microbial presence. Each TLR recognises specific PAMPs: TLR9 detects unmethylated CpG DNA motifs; TLR7 recognises single-stranded RNA; TLR4 responds to lipopolysaccharide; and TLR2 senses bacterial lipopeptides and lipoglycans [67,68]. Dual engagement of the BCR and TLRs dramatically lowers activation thresholds—a phenomenon termed BCR-TLR synergy [69].

TLR7 gain-of-function variants cause monogenic lupus, with a Y99H variant identified as sufficient to drive SLE in both humans and mice [70]. TLR7 gene duplication increases lupus risk, potentially contributing to the female predominance through gene dosage effects [71].

### 4.2. Type I Interferon: The Master Amplifier

Type I interferons emerge as master amplifiers of plasmablast responses. pDCs serve as the principal IFN-I producers, secreting 1000-fold more IFN-alpha than other cell types upon TLR7 or TLR9 engagement [72]. IFN-I drives plasmablast differentiation through induction of IRF4 and BLIMP-1, and upregulates TLR7 expression on B cells, creating positive feedback [73]. The IFN signature is present in 50–80% of active SLE patients. Therapeutic targeting with anifrolumab has demonstrated efficacy, validating this pathway [74].

Similar signatures have been identified in subsets of patients with Sjogren syndrome, dermatomyositis, and systemic sclerosis. From the plasmablast perspective, IFN-I functions as the inflammatory context that licenses extrafollicular B-cell responses to proceed unchecked.

### 4.3. Cytokine Networks Supporting Plasmablast Differentiation

IL-6 is indispensable for plasma cell differentiation, acting through STAT3 to induce BLIMP-1 [75]. IL-21, produced by Tfh cells, potently drives B-cell proliferation and plasmablast differentiation [76]. Microbial triggers that activate multiple arms of this cytokine network simultaneously generate particularly robust plasmablast responses [77].

## 5. Microbiome Dysbiosis and B-Cell Activation

### 5.1. The Gut as a Chronic, Low-Grade Microbial Stimulus

The intestinal microbiota, comprising trillions of bacteria, fungi, and viruses, profoundly shapes immune development and homeostasis. Dysbiosis, pathological alterations in microbiome composition, has been documented across autoimmune conditions including SLE, RA, Sjogren syndrome, and inflammatory myopathies. While establishing causality remains challenging, experimental models demonstrate that microbiome manipulation can modulate autoimmune phenotypes, and emerging human data support mechanistic connections.

From the plasmablast storm perspective, the gut microbiome functions as a chronic, low-grade source of microbial stimulation that keeps B cells poised near activation thresholds, thereby lowering the stimulus required for discrete triggers to ignite full-blown plasmablast responses. Unlike acute infections that provide transient, high-intensity signals, dysbiosis creates a persistent state of immune priming [78]. This tonic stimulation may not be sufficient to trigger overt plasmablast storms independently but sensitises the B-cell compartment such that subsequent viral reactivations, acute infections, or other triggers more readily precipitate autoreactive responses. The gut-immune axis influences B-cell biology through multiple mechanisms. Intestinal epithelial cells and dendritic cells sense microbial products through pattern recognition receptors, producing cytokines including BAFF, APRIL, and IL-6 that can act systemically to modulate B-cell survival and differentiation. Gut-associated lymphoid tissue (GALT), including Peyer patches and isolated lymphoid follicles, serves as a major site of B-cell activation, class switching, and IgA production. Perturbations in intestinal homeostasis can alter the output of these responses, potentially seeding systemic autoimmunity.

Specific bacterial taxa have been associated with autoimmune disease. Ruminococcus gnavus, expanded in active SLE, produces lipoglycans that stimulate dendritic cells via TLR2 (demonstrated in human cell culture and murine models) [79]. Enterococcus gallinarum translocates across the gut barrier in lupus-prone mice and has been detected in liver tissue of SLE patients, inducing autoantibody production through type I interferon pathways [80].

### 5.2. Metabolite-Mediated B-Cell Modulation

Microbiota-derived metabolites represent a key mechanism through which dysbiosis influences systemic immunity and B-cell function. SCFAs, including acetate, propionate, and butyrate, are produced by bacterial fermentation of dietary fibre and exert dose-dependent effects on immune cells. Butyrate inhibits histone deacetylases (HDACs), enabling epigenetic modulation of gene expression, and can suppress B-cell class switching and antibody production through effects on AID expression (established in murine models and human B-cell cultures) [81]. Reduced SCFA-producing bacteria, commonly observed in autoimmune dysbiosis, may thus release B cells from metabolic restraint.

The mechanistic chain from dysbiosis to plasmablast permissiveness operates through defined molecular steps. Loss of SCFA-producing taxa removes butyrate-mediated HDAC inhibition. Reduced butyrate releases epigenetic brakes on plasmablast differentiation genes, lowering the threshold for IRF4 and BLIMP-1 induction [82].

While experimental models strongly support a role for microbial metabolites in shaping B-cell differentiation thresholds, direct causal evidence in humans remains limited. Most human studies demonstrate associative links between dysbiosis, altered metabolite profiles (e.g., reduced short-chain fatty acids), and autoimmune disease activity.

The effects of SCFAs on B-cell differentiation have been demonstrated in vitro and in murine models, but direct causal evidence in human autoimmune disease remains limited to correlational observations (reduced SCFA-producing bacteria in patient cohorts, inverse correlations between faecal butyrate and disease activity). Similarly, while tryptophan metabolite shifts have been documented in SLE and RA patients, whether these represent drivers or consequences of inflammation requires interventional studies. Ongoing clinical trials of dietary fibre supplementation and defined bacterial consortia will help establish which microbiome-plasmablast connections are therapeutically tractable in humans.

At present, it remains unresolved whether metabolite alterations act as primary drivers of plasmablast differentiation in humans or function predominantly as permissive modulators that lower activation thresholds in genetically predisposed hosts. Accordingly, microbiome-derived metabolite effects should be interpreted as context-setting rather than deterministic, pending interventional validation in human studies.

### 5.3. Oral-Gut-Joint Axis in Rheumatoid Arthritis

Prevotella copri expansion in new-onset RA represents one of the most replicated microbiome findings in autoimmunity [83]. Prevotella-derived metabolites may promote Th17 differentiation and joint inflammation [84].

### 5.4. Barrier Dysfunction and Microbial Translocation

Intestinal barrier dysfunction, or ‘leaky gut,’ permits translocation of bacterial products into the systemic circulation [85]. SLE patients show elevated serum lipopolysaccharide (LPS) and correlations between microbial translocation markers and autoantibody levels have been reported [86].

## 6. T-Cell Cooperation and Tissue-Level Inflammation

While extrafollicular plasmablast responses can proceed with reduced T-cell dependence relative to germinal centre reactions, T-cell help remains important for sustaining and amplifying these responses. Peripheral helper T cells (Tph), distinct from classical Tfh cells, have been identified in inflamed tissues of patients with RA and other autoimmune conditions [87]. Tph cells express high levels of PD-1 and produce IL-21, providing B-cell help in ectopic lymphoid structures [88].

Microbial triggers can expand these T-cell populations through bystander activation and cytokine-driven proliferation, further amplifying the capacity for plasmablast storms.

## 7. Plasmablast Trafficking and Tissue Homing

### Chemokine Guidance of Plasmablast Migration

Plasmablasts express distinct chemokine receptor profiles that determine their migratory behaviour and tissue localisation. CXCR4, the receptor for CXCL12 (SDF-1), guides plasmablasts toward bone marrow niches where they may mature into long-lived plasma cells [89]. In contrast, CXCR3, which binds CXCL9, CXCL10, and CXCL11, directs plasmablasts toward inflamed tissues expressing these interferon-induced chemokines. Microbial triggers shape this chemokine landscape through induction of IFN-I and inflammatory cytokines, determining whether circulating plasmablasts seed bone marrow long-term reservoirs or home to inflamed organs where they mediate local antibody-dependent damage. The balance between CXCR3 and CXCR4 expression on individual plasmablasts thus influences the anatomical distribution and persistence of autoantibody production. In active SLE, plasmablasts demonstrate enhanced CXCR3 expression, correlating with renal and cutaneous manifestations [90]. Inflamed kidneys in lupus nephritis upregulate CXCL10, creating chemotactic gradients that recruit CXCR3^+^ plasmablasts to sites of tissue injury. This tissue-directed homing amplifies local autoantibody deposition and immune complex formation.

The chemokine receptor repertoire of plasmablasts is dynamically regulated during differentiation. Early plasmablasts express high levels of CXCR3, facilitating rapid tissue infiltration, while maturing cells progressively upregulate CXCR4, enabling bone marrow homing [91]. Inflammatory cytokines, particularly IFN-I and IL-6, can arrest this developmental programme, maintaining CXCR3 expression and prolonging tissue residence.

## 8. Autoantibody Diversification and Epitope Spreading

### 8.1. Plasmablast-Driven Repertoire Evolution

A critical consequence of repeated plasmablast storms is progressive diversification of the autoantibody repertoire [92]. While individual plasmablast bursts are often oligoclonal, dominated by expanded clones responding to acute triggers, cumulative episodes drive epitope spreading and affinity maturation that entrench autoimmunity. Analysis of longitudinal patient samples reveals that autoantibody specificities broaden over disease duration, with new epitope reactivities emerging during flares and often persisting into remission [93].

Somatic hypermutation (SHM) within extrafollicular responses, though less extensive than in germinal centres, nonetheless contributes to antibody diversification [94]. IFN-I and TLR signalling can induce activation-induced cytidine deaminase (AID), the enzyme responsible for SHM and class-switch recombination, in extrafollicular B cells. This enables ongoing antibody diversification outside classical GC structures, potentially generating higher-affinity autoreactive variants during successive plasmablast storms. BCR sequencing studies in SLE patients demonstrate clonal evolution with accumulating mutations over time [95].

### 8.2. Epitope Spreading and Tolerance Erosion

Epitope spreading, the extension of immune responses from initial target epitopes to additional epitopes on the same or related antigens, is a hallmark of progressive autoimmunity [96]. In SLE, responses may begin against a single component of ribonucleoprotein complexes (e.g., SmB) and spread to additional components (SmD, RNP-A, RNP-C) over time. Studies of pre-clinical lupus demonstrate sequential appearance of autoantibody specificities, with anti-Ro often preceding anti-La and anti-DNA responses [97]. ACPA responses in RA similarly diversify from initial citrullinated targets to encompass multiple citrullinated proteins.

Plasmablast storms contribute to epitope spreading through several mechanisms. First, inflammatory tissue damage releases intracellular and sequestered antigens, exposing new epitopes to the immune system [98]. Second, SHM introduces mutations that may broaden BCR cross-reactivity. Third, the inflammatory cytokine milieu during flares disrupts peripheral tolerance mechanisms that normally restrain autoreactive B cells.

Each microbial trigger thus creates not only an immediate plasmablast storm but also an opportunity for repertoire diversification that progressively erodes tolerance and stabilises pathogenic B-cell clones [99].

### 8.3. Memory B Cells and Long-Lived Plasma Cells

While plasmablasts themselves are short-lived, the B-cell responses they emerge from generate memory populations that enable rapid recall during subsequent triggers [100]. Memory B cells arising from extrafollicular responses may carry autoreactive specificities that permit immediate plasmablast differentiation upon re-encounter with self-antigens or during subsequent microbial triggers that create permissive inflammatory contexts. Longitudinal studies of SLE patients reveal persistence of autoreactive B-cell clones across years, with clone frequencies expanding during flares and contracting during remission [101].

A proportion of plasmablasts also mature into long-lived plasma cells (LLPCs) that seed bone marrow niches, establishing durable autoantibody production independent of ongoing B-cell activation [102]. LLPCs can persist for decades and are resistant to conventional immunosuppression and B-cell depletion therapies. The interplay between acute plasmablast storms and these long-term memory and plasma cell populations creates the complex serological dynamics characteristic of chronic autoimmunity. Targeting LLPCs may be necessary for achieving true immunological remission [103].

## 9. Clinical Implications and Therapeutic Opportunities

### 9.1. Plasmablasts as Disease Biomarkers

Circulating plasmablast frequencies correlate with disease activity across multiple autoimmune conditions, positioning them as potential biomarkers for monitoring and predicting flares [40]. In SLE, plasmablast percentages above 2–3% of peripheral B cells strongly associate with active disease, particularly nephritis. Flow cytometric enumeration of CD19^low^CD27^high^CD38^high^ cells provides a readily implementable assay for clinical practice [104]. Rising plasmablast counts may herald impending flares before clinical symptoms manifest, enabling pre-emptive therapeutic intensification.

Beyond enumeration, molecular characterisation of plasmablasts offers additional insights. Single-cell RNA sequencing and BCR repertoire analysis can reveal clonal dynamics, identify disease-relevant specificities, and track responses to therapy [105]. The oligoclonal nature of many plasmablast expansions suggests that specific clones drive individual flares, potentially enabling targeted intervention against pathogenic specificities. Plasmablast-derived autoantibody profiles may also inform prognosis and guide therapeutic selection.

### 9.2. Viral Monitoring and Antiviral Strategies

Given the strong associations between herpesvirus reactivation and autoimmune flares, monitoring EBV and CMV viral loads or serological markers may identify patients at risk for reactivation-associated plasmablast surges [47]. Serial measurement of EBV DNA by quantitative PCR could potentially predict impending flares in patients with established SLE or Sjogren syndrome, enabling pre-emptive immunosuppressive adjustment. Similarly, CMV antigenaemia or DNA monitoring, already standard practice in transplant medicine, might have applications in heavily immunosuppressed autoimmune patients at high risk for CMV-triggered disease exacerbations.

Antiviral prophylaxis or pre-emptive treatment represents a theoretical but largely unexplored strategy to blunt microbe-licensed plasmablast storms in high-risk patients. Acyclovir and valacyclovir suppress herpesvirus replication and could, in principle, reduce the frequency of reactivation-triggered flares, particularly in heavily immunosuppressed patients receiving rituximab, cyclophosphamide, or other lymphocyte-depleting therapies who are at increased risk for herpesvirus reactivation [106]. However, clinical trial data supporting this approach in autoimmune disease are lacking, and the cost-effectiveness of universal prophylaxis is uncertain. A more targeted approach might involve viral monitoring to identify patients with rising EBV or CMV loads, followed by pre-emptive antiviral therapy before clinical flare develops. This strategy remains investigational and requires prospective validation. Integrated biomarker panels combining plasmablast enumeration with viral load monitoring may enhance flare prediction. Preliminary data from lupus cohorts suggest that rising EBV DNA titres preceding plasmablast expansion could identify a ‘pre-storm’ window amenable to pre-emptive intervention. A proposed monitoring panel includes: (1) plasmablast frequency by flow cytometry (CD19^low^CD27^high^CD38^high^); (2) EBV/CMV viral loads by quantitative PCR; (3) IFN-signature score (gene expression or serum protein panel); and (4) disease-relevant autoantibody titres. Prospective studies evaluating whether this combined approach outperforms single biomarkers for flare prediction are warranted.

### 9.3. Targeting Innate Sensing Pathways

Therapeutic blockade of innate sensing pathways offers a strategy for preventing the inflammatory contexts that license plasmablast storms (Table 1). Anifrolumab, a monoclonal antibody blocking the type I IFN receptor (IFNAR1), has demonstrated efficacy in moderate-to-severe SLE and represents proof of concept for this approach [107,108]. By neutralising IFN-I signalling regardless of whether it originates from viral infection, immune complex stimulation, or other sources, anifrolumab reduces B-cell activation signals, diminishes plasmablast differentiation, and lowers autoantibody titres. The selective benefit in IFN-high patients highlights the importance of stratifying patients by pathway activation.

TLR7 and TLR9 inhibitors are in clinical development for autoimmune indications. Enpatoran (M5049), a dual TLR7/8 inhibitor, has shown promising results in early lupus trials [109]. Given the central role of these receptors in sensing nucleic acid-containing immune complexes and microbial PAMPs, their inhibition may directly restrain plasmablast responses to both endogenous triggers and exogenous microbial stimuli. JAK inhibitors, which block signalling downstream of IFN receptors and multiple cytokine receptors, have shown efficacy in RA and SLE and may exert part of their benefit through dampening plasmablast-permissive inflammation [110].

### 9.4. B-Cell Directed Therapies

Direct targeting of B-cell lineage cells remains a cornerstone of autoimmune therapy. Rituximab, depleting CD20+ B cells, effectively eliminates precursors capable of generating plasmablasts while sparing mature plasma cells that lack CD20 (Table 2) [111]. The clinical observation that rituximab responses are often delayed and may require multiple cycles is consistent with gradual depletion of autoreactive memory populations that seed plasmablast storms. Variable responses across patients and conditions may reflect differences in the contribution of B-cell-dependent versus plasma cell-dependent autoantibody production.

Belimumab, blocking BAFF, reduces B-cell survival signals and modestly diminishes plasmablast frequencies over time [111,112]. Combination of belimumab with rituximab has shown enhanced efficacy in lupus nephritis, suggesting synergy between survival signal blockade and B-cell depletion. Obinutuzumab, a glycoengineered anti-CD20 antibody with enhanced antibody-dependent cellular cytotoxicity, has demonstrated superiority to rituximab in lupus nephritis trials [113].

More recently, CD19-directed therapies, including the bispecific T-cell engager blinatumomab and CD19 CAR-T cells, have shown remarkable efficacy in refractory autoimmunity, achieving deep B-cell depletion that encompasses both CD20^+^ B cells and CD20^−^ plasmablasts [114,115]. Early reports suggest potential for drug-free remission following B-cell reconstitution, consistent with elimination of pathogenic clones and immune reset. The durability of these responses and long-term safety require further investigation, but they provide proof of concept that elimination of autoreactive B-cell populations can fundamentally alter disease trajectory.

CD19-directed chimeric antigen receptor T-cell (CAR-T) therapy has emerged as a transformative approach for refractory autoimmune disease. The landmark study by Mackensen et al. (2022) demonstrated drug-free remissions in five patients with refractory SLE following CD19 CAR-T infusion [116]. Subsequent reports have extended these findings to antisynthetase syndrome, systemic sclerosis, and other refractory conditions [116,117,118,119,120]. The mechanism involves deep B-cell depletion encompassing both CD20+ B cells and CD20− plasmablasts/plasma cells, achieving a degree of B-lineage elimination not possible with rituximab. Updated follow-up data (2024) demonstrate sustained remissions exceeding 24 months in most patients, with B-cell reconstitution occurring from naive precursors apparently purged of autoreactive memory. Ongoing trials (NCT05765006, NCT05030779) are evaluating CAR-T in broader autoimmune populations and optimising conditioning regimens.

Bispecific antibodies engaging T cells to eliminate B-lineage cells represent an ‘off-the-shelf’ alternative to CAR-T. CD19 × CD3 bispecifics (blinatumomab) and CD20 × CD3 bispecifics (mosunetuzumab, glofitamab) are approved for B-cell malignancies and under investigation in autoimmunity. Potential advantages include immediate availability, titratable dosing, and avoidance of lymphodepletion conditioning. BCMA-targeting approaches, originally developed for myeloma, may enable selective depletion of long-lived plasma cells responsible for persistent autoantibody production [116,119].

### 9.5. Microbiome-Targeted Interventions

The recognition of microbiome contributions to autoimmune pathogenesis has generated interest in microbiome-targeted therapeutics [121]. Probiotics, prebiotics, faecal microbiota transplantation (FMT), and defined bacterial consortia are under investigation, though clinical evidence in autoimmune disease remains preliminary. From the plasmablast perspective, successful microbiome modulation could reduce circulating microbial products, restore SCFA production, and dampen the tonic inflammation that primes B cells for extrafollicular responses.

The mechanistic rationale for microbiome-targeted therapy in plasmablast-driven disease centres on reducing tonic microbial TLR signalling and restoring metabolic restraints on B-cell differentiation. Dietary interventions, particularly those increasing fibre intake to support SCFA-producing bacteria, may restore butyrate-mediated HDAC inhibition and thereby raise the threshold for B-cell activation and plasmablast differentiation. FMT or defined consortia enriched in SCFA-producers could achieve similar effects. Strategies to repair barrier integrity, including glutamine supplementation, zinc, and probiotics with demonstrated effects on tight junction proteins, could reduce translocation of LPS, bacterial DNA, and other PAMPs that provide ongoing TLR stimulation to circulating B cells [121]. While diet and microbiome modulation alone are unlikely to induce remission in established autoimmunity, they may reduce flare frequency by keeping B cells further from activation thresholds, such that stronger triggers are required to ignite plasmablast storms [122,123]. Microbiome engineering approaches under investigation include: (1) Live biotherapeutic products (LBPs) comprising defined bacterial consortia designed to restore eubiosis (e.g., VE303, SER-287); (2) Precision prebiotics selectively promoting beneficial taxa; (3) Phage therapy targeting specific pathobionts such as Ruminococcus gnavus or Enterococcus gallinarum; and (4) Barrier repair strategies using glutamine, zinc, or probiotics with demonstrated effects on tight junction integrity.

### 9.6. Plasmablast Storms Across Disease Contexts

While the plasmablast storm model was initially developed from SLE research, emerging evidence reveals both shared features and disease-specific patterns across autoimmune conditions:

Systemic lupus erythematosus: Characterized by dominant extrafollicular pathway activation, ISG^high^ plasmablast signatures, and autoantibodies targeting nuclear antigens (dsDNA, Sm, RNP). Plasmablast frequencies correlate strongly with nephritis activity and predict renal flares. EBV reactivation is a well-documented trigger [122].

Rheumatoid arthritis: ACPA-secreting plasmablasts arise through the ‘mouth-to-flare’ pathway involving periodontal pathogens. Synovial ectopic lymphoid structures harbour local plasmablast generation. Unlike SLE, IFN-signature is less prominent; IL-6-driven pathways may predominate [124].

Sjögren syndrome: Salivary gland-resident plasmablasts producing anti-Ro/La antibodies are characteristic. Strong EBV association with elevated viral loads in salivary tissue. Risk of B-cell lymphoma transformation reflects chronic B-cell stimulation [125].

ANCA-associated vasculitis: Anti-PR3 and anti-MPO plasmablasts drive disease. Excellent response to rituximab supports central role of B-cell lineage. Early CAR-T data show promise in refractory cases [126].

Inflammatory myopathies: Anti-synthetase and anti-MDA5 antibody-secreting plasmablasts expanded during active disease. Strong IFN-signature parallels SLE. Viral triggers (including SARS-CoV-2) documented as precipitants [127,128].

## 10. Future Directions and Research Priorities

Several key questions warrant investigation to translate the plasmablast storm model into improved patient outcomes. First, prospective studies with intensive longitudinal sampling are needed to define the temporal dynamics of plasmablast responses relative to microbial triggers, clinical symptoms, and serological changes [128]. Integration of viral monitoring (EBV, CMV loads), microbiome profiling, and plasmablast enumeration could identify the relative contributions of different microbial inputs to individual flares and enable biomarker-guided pre-emptive therapy.

Second, deeper characterisation of plasmablast populations arising during flares, through single-cell technologies, BCR sequencing, and functional assays, will clarify which clones drive pathology and whether specific microbial triggers selectively expand certain autoreactive specificities [129]. This knowledge could inform development of antigen-specific tolerogenic strategies or selective depletion approaches that spare protective immunity while eliminating pathogenic clones.

Third, the interplay between chronic dysbiosis and acute microbial triggers deserves mechanistic investigation. Does restoration of eubiosis raise the threshold for viral reactivation-triggered flares? Can barrier repair strategies reduce the frequency of plasmablast storms even without eliminating the underlying autoimmune diathesis? [83] Interventional studies combining microbiome modulation with standard immunosuppression could address these questions.

Fourth, combination therapeutic strategies targeting multiple nodes of the plasmablast storm pathway merit exploration. The redundancy inherent in immune activation suggests that single-target approaches may have ceiling effects, while combinations targeting innate sensing (TLR inhibition, IFN-I blockade), cytokine signalling (JAK inhibition), and B-cell survival (BAFF blockade, CD20/CD19 depletion) may achieve synergistic dampening of autoreactive responses [130]. Rational combination design guided by mechanistic understanding could improve efficacy while managing safety considerations.

Emerging technologies will transform our understanding of plasmablast biology and tissue organisation. Spatial transcriptomics enables mapping of plasmablast niches within inflamed tissues, identifying stromal support cells and understanding the architecture of ectopic lymphoid structures. Recent spatial transcriptomic analysis of lupus nephritis demonstrated plasmablast clustering near damaged tubular epithelium, suggesting direct antibody-mediated injury mechanisms [131]. Single-cell multi-omics approaches combining scRNA-seq, BCR sequencing, and surface proteomics (CITE-seq) can simultaneously profile transcriptome, B-cell receptor repertoire, and phenotype, enabling clonal tracking of autoreactive plasmablasts from memory precursors through storm expansion. CRISPR-based functional genomic screens in primary human B cells are beginning to identify genes essential for plasmablast differentiation in inflammatory contexts, revealing potential novel therapeutic targets [132,133].

## 11. Limitations of the Plasmablast Storm Model

Several limitations of the plasmablast storm framework warrant explicit acknowledgment. Not all flares involve plasmablast storms. Some disease exacerbations may be driven primarily by T-cell-mediated mechanisms, complement activation, or innate immune pathways without prominent B-cell involvement. Seronegative disease subsets and flares occurring during effective B-cell depletion therapy illustrate that plasmablast-independent mechanisms exist.

Furthermore, evidence linking microbial triggers to plasmablast expansion remains associative rather than causal in humans. There is limited proofs that antiviral prophylaxis prevents flares or that microbiome restoration reduces plasmablast storms. Inter-individual variability in microbial exposure, genetics, and immune regulation likely determines whether plasmablast storms translate into clinical flares. Accordingly, plasmablast storms should be viewed as a dominant but not exclusive driver of autoimmune flares.

The model does not fully explain why identical microbial exposures trigger plasmablast storms in some individuals but not others. HLA associations, TLR polymorphisms, and B-cell intrinsic factors likely determine susceptibility, but integration of host genetics into the framework requires further development.

In addition, the model is best developed for systemic autoimmune diseases with circulating plasmablasts; application to organ-specific autoimmunity (e.g., type 1 diabetes, autoimmune thyroiditis) where plasmablasts may be tissue-restricted requires additional validation.

The temporal resolution of the plasmablast storm cascade remains incompletely defined. The precise kinetics linking microbial exposure, innate immune activation, plasmablast expansion, and clinical flare onset are not yet fully delineated. High-resolution longitudinal studies with frequent sampling of viral load, cytokine signatures, and B-cell subsets will be required to identify predictive windows for pre-emptive intervention.

## 12. Conclusions

Plasmablast storms represent a unifying concept bridging microbial immunology with autoimmune pathophysiology. Three distinct categories of microbial input, latent herpesvirus reactivation, acute respiratory/gastrointestinal infection, and chronic microbiome dysbiosis, converge on innate sensing pathways through specific PAMPs, including CpG DNA, ssRNA, LPS, and bacterial lipoglycans. TLR7/9 engagement, particularly through BCR-TLR synergy, and type I interferon signalling create inflammatory contexts permissive for rapid, extrafollicular B-cell responses that bypass normal tolerance checkpoints.

The recurrent nature of these plasmablast storms shapes the natural history of autoimmune disease. Each acute expansion drives autoantibody diversification through somatic hypermutation and promotes epitope spreading through the release of sequestered antigens and disruption of peripheral tolerance. This progressive erosion of tolerance checkpoints establishes a vicious cycle whereby successive flares entrench autoimmunity and generate the long-lived memory B cells and plasma cells that sustain chronic disease. Chronic dysbiosis and barrier dysfunction maintain B cells near activation thresholds, creating ongoing vulnerability to future storms triggered by viral reactivation or acute infection.

Therapeutic opportunities emerge at multiple levels of this framework. Antiviral strategies may blunt herpesvirus-triggered storms in high-risk patients. Targeting innate amplification through IFN-I blockade (Anifrolumab) or TLR inhibition can prevent the inflammatory licensing of plasmablast responses. B-cell-directed therapies can eliminate precursors and pathogenic clones. Modulation of cytokine networks through JAK inhibition or IL-6 blockade dampens the signals driving plasmablast differentiation. Microbiome modulation may reduce tonic TLR stimulation and restore metabolic restraints on B-cell differentiation. By understanding plasmablast storms as the nexus where diverse microbial inputs ignite autoimmunity, we gain leverage for interventions that interrupt this cycle and modify the trajectory of chronic autoimmune disease.

## Figures and Tables

**Table 1:** Microbial Inputs, Dominant PAMPs, and Plasmablast Storm Outcomes.

Microbial Trigger	Dominant PAMPs	Main Sensing Pathway	Typical Outcome
EBV reactivation	CpG DNA, EBER RNAs, LMP1 protein	TLR9, TLR7, TLR2; pDC IFN-I	Oligoclonal plasmablast storm; anti-dsDNA, anti-Sm/RNP cross-reactivity
CMV reactivation	CpG DNA, dsDNA, glycoproteins	TLR9, cGAS-STING, TLR2; IFN-I surge	Bystander B-cell activation; CD4+CD28null expansion; immunosenescence
SARS-CoV-2 infection	ssRNA, spike protein	TLR7, TLR8, RIG-I/MDA5; massive IFN-I	EF-dominant plasmablast storm; autoantibodies in 10–50%
Influenza infection	ssRNA, HA protein	TLR7, RIG-I; transient IFN-I	Transient plasmablast burst; occasional autoantibody induction
P. gingivalis (periodontitis)	LPS, lipoproteins, PAD enzymes	TLR2, TLR4; citrullination of host proteins	ACPA-secreting plasmablasts; RA flare via ‘mouth-to-flare’ pathway
Gut dysbiosis (chronic)	LPS, CpG DNA, lipoglycans (R. gnavus)	TLR4, TLR9, TLR2; tonic activation	B-cell priming; lowered threshold for plasmablast storms
E. gallinarum translocation	Bacterial DNA, cell wall components	TLR7/9, AhR; IFN-I induction	Systemic autoantibody production; lupus-like disease
SCFA depletion (metabolite shift)	Loss of butyrate, propionate	HDAC de-repression; epigenetic priming	Lowered IRF4/BLIMP-1 threshold; enhanced plasmablast differentiation

Abbreviations: ACPA, anti-citrullinated protein antibody; AhR, aryl hydrocarbon receptor; CpG, cytosine-phosphate-guanine; dsDNA, double-stranded DNA; EBER, Epstein–Barr virus-encoded small RNA; EF, extrafollicular; HA, haemagglutinin; HDAC, histone deacetylase; IFN-I, type I interferon; LPS, lipopolysaccharide; MDA5, melanoma differentiation-associated protein 5; PAD, peptidylarginine deiminase; PAMP, pathogen-associated molecular pattern; pDC, plasmacytoid dendritic cell; RA, rheumatoid arthritis; RIG-I, retinoic acid-inducible gene I; SCFA, short-chain fatty acid; ssRNA, single-stranded RNA; STING, stimulator of interferon genes; TLR, Toll-like receptor.

**Table 2:** Therapeutic Strategies Targeting Plasmablast Storms in Autoimmune Disease.

Target	Agent(s)	Mechanism	Clinical Status
Type I IFN receptor	Anifrolumab	Blocks IFNAR1; reduces ISG expression, B-cell activation, plasmablast differentiation	FDA-approved for SLE
TLR7/TLR8	Enpatoran (M5049)	Inhibits sensing of viral ssRNA, bacterial RNA; reduces BCR-TLR synergy	Phase II trials in SLE, CLE
JAK1/JAK2	Baricitinib, upadacitinib	Blocks IFN-I/IL-6/IL-21 signalling downstream of cytokine receptors	Approved for RA; Phase III in SLE
BAFF	Belimumab	Neutralises B-cell survival factor; reduces transitional/naive B cells, plasmablasts	FDA-approved for SLE, LN
CD20	Rituximab, obinutuzumab	Depletes CD20+ B cells; eliminates plasmablast precursors and memory B cells	Approved for RA, AAV; obinutuzumab approved for LN
CD19	CD19 CAR-T, blinatumomab	Deep B-cell depletion including plasmablasts; potential immune reset	Investigational; promising early results
IL-6 receptor	Tocilizumab, sarilumab	Blocks IL-6 signalling; reduces STAT3-driven plasma cell differentiation	Approved for RA, GCA; trials in SSc
Herpesviruses	Valacyclovir, valganciclovir	Suppresses EBV/CMV reactivation; reduces viral PAMP release and IFN-I trigger	Investigational in autoimmunity
Microbiome/Barrier	FMT, probiotics, dietary fibre	Restores SCFA; reduces microbial translocation and tonic TLR signalling to B cells	Early clinical investigation

Abbreviations: AAV, ANCA-associated vasculitis; BAFF, B-cell activating factor; CAR-T, chimeric antigen receptor T cell; CLE, cutaneous lupus erythematosus; CMV, cytomegalovirus; EBV, Epstein–Barr virus; FMT, faecal microbiota transplantation; GCA, giant cell arteritis; IFN, interferon; IFNAR, interferon-alpha/beta receptor; ISG, interferon-stimulated gene; JAK, Janus kinase; LN, lupus nephritis; PAMP, pathogen-associated molecular pattern; pDC, plasmacytoid dendritic cell; RA, rheumatoid arthritis; SCFA, short-chain fatty acid; SLE, systemic lupus erythematosus; SSc, systemic sclerosis; TLR, Toll-like receptor.

## Data Availability

No new data were created or analyzed in this study. Data sharing is not applicable to this article.

## List of Abbreviations

ACPA	Anti-citrullinated protein antibodies
ANA	Antinuclear antibodies
ANCA	Anti-neutrophil cytoplasmic antibodies
APRIL	A proliferation-inducing ligand
ASC	Antibody-secreting cell
BAFF	B-cell activating factor
BCR	B-cell receptor
BLIMP-1	B lymphocyte-induced maturation protein-1
CAR-T	Chimeric antigen receptor T cell
CD	Cluster of differentiation
CITE-seq	Cellular indexing of transcriptomes and epitopes by sequencing
CMV	Cytomegalovirus
COVID-19	Coronavirus disease 2019
CpG	Cytosine-phosphate-guanine
CRISPR	Clustered regularly interspaced short palindromic repeats
CXCR	C-X-C chemokine receptor
DNA	Deoxyribonucleic acid
DORIS	Definition of remission in SLE
dsDNA	Double-stranded deoxyribonucleic acid
EBV	Epstein–Barr virus
EF	Extrafollicular
GC	Germinal centre
HDAC	Histone deacetylase
HHV-6	Human herpesvirus 6
HLA	Human leukocyte antigen
IFN	Interferon
IFN-I	Type I interferon
IFN-α	Interferon-alpha
Ig	Immunoglobulin
IgG	Immunoglobulin G
IL	Interleukin
IL-6	Interleukin-6
IL-21	Interleukin-21
IRF4	Interferon regulatory factor 4
ISG	Interferon-stimulated gene
JAK	Janus kinase
LPS	Lipopolysaccharide
MDA5	Melanoma differentiation-associated protein 5
MPO	Myeloperoxidase
NET	Neutrophil extracellular trap
NETosis	Neutrophil extracellular trap formation
PAD	Peptidylarginine deiminase
PAMP	Pathogen-associated molecular pattern
pDC	Plasmacytoid dendritic cell
PR3	Proteinase 3
PRDM1	PR domain zinc finger protein 1
RA	Rheumatoid arthritis
RNA	Ribonucleic acid
RNP	Ribonucleoprotein
SARS-CoV-2	Severe acute respiratory syndrome coronavirus 2
SCFA	Short-chain fatty acid
scRNA-seq	Single-cell RNA sequencing
SLE	Systemic lupus erythematosus
SLEDAI	Systemic Lupus Erythematosus Disease Activity Index
Sm	Smith antigen
STAT	Signal transducer and activator of transcription
STAT3	Signal transducer and activator of transcription 3
Tfh	T follicular helper
TLR	Toll-like receptor
TLR2	Toll-like receptor 2
TLR4	Toll-like receptor 4
TLR7	Toll-like receptor 7
TLR8	Toll-like receptor 8
TLR9	Toll-like receptor 9
TYK2	Tyrosine kinase 2
VZV	Varicella-zoster virus
